# Impaired microRNA processing by DICER1 downregulation endows thyroid cancer with increased aggressiveness

**DOI:** 10.1038/s41388-019-0804-8

**Published:** 2019-04-09

**Authors:** Julia Ramírez-Moya, León Wert-Lamas, Garcilaso Riesco-Eizaguirre, Pilar Santisteban

**Affiliations:** 1 Instituto de Investigaciones Biomédicas “Alberto Sols”; Consejo Superior de Investigaciones Científicas (CSIC), Universidad Autónoma de Madrid (UAM), Madrid, Spain; 2 Centro de Investigación Biomédica en Red de Cáncer (CIBERONC), Instituto de Salud Carlos III (ISCIII), Madrid, Spain; 3 Departamento de Endocrinología y Nutrición, Hospital Universitario de Móstoles, Madrid, Spain; 4 Universidad Francisco de Vitoria, Madrid, Spain

**Keywords:** Thyroid cancer, Non-coding RNAs

## Abstract

The global downregulation of microRNAs (miRNAs) is emerging as a common hallmark of cancer. However, the mechanisms underlying this phenomenon are not well known. We identified that the oncogenic miR-146b-5p attenuates miRNA biosynthesis by targeting DICER1 and reducing its expression. DICER1 overexpression inhibited all the miR-146b-induced aggressive phenotypes in thyroid cells. Systemic injection of an anti-miR-146b in mice with orthotopic thyroid tumors suppressed tumor growth and recovered DICER1 levels. Notably, DICER1 downregulation promoted proliferation, migration, invasion, and epithelial-mesenchymal transition through miRNA downregulation. Our analysis of The Cancer Genome Atlas revealed a general decrease in DICER1 expression in thyroid cancer that was associated with a worse clinical outcome. Administration of the small-molecule enoxacin to promote DICER1 complex activity reduced tumor aggressiveness both in vitro and in vivo. Overall, our data confirm DICER1 as a tumor suppressor and show that oncogenic miR-146b contributes to its downregulation. Moreover, our results highlight a potential therapeutic application of RNA-based therapies including miRNA inhibitors and restoration of the biogenesis machinery, which may provide treatments for thyroid and other cancers.

## Introduction

MicoRNAs (miRNAs) are short noncoding RNAs that function post-transcriptionally to suppress gene expression through interactions of their seed region with complementary sequences in the 3′-untranslated regions (UTRs) of target messenger RNAs, which consequently affects a myriad of cellular and developmental pathways [1, 2]. Recently, miRNAs have taken center stage in molecular oncology due to their participation in the development and progression of human neoplasms, including thyroid tumors [3, 4].

Thyroid cancer is the most frequent endocrine malignancy and is the most rapidly increasing of all cancers in the United States [5]. The classical view of thyroid cancer pathogenesis considers thyroid carcinomas as tumors accumulating mutations that drive progression through a dedifferentiation process initially giving rise to the well-differentiated carcinomas—papillary (PTC) and follicular (FTC)—and progressing to poorly differentiated (PDTC) and undifferentiated or anaplastic (ATC) thyroid carcinomas [6]. Activation of oncogenes is a known cause of miRNA deregulation in thyroid cells. Among the most well-studied oncogenes that drive malignancy in thyroid tumors are BRAF, RAS, and RET/PTC, which induce a distinct set of global changes in the expression of miRNAs [7]. Genomic analysis of PTC tumors in The Cancer Genome Atlas (TCGA) suggests that miRNA expression patterns define clinically relevant subclasses and may contribute to loss of differentiation and tumor progression [3, 8]. Several studies have shown that miRNA expression is globally suppressed in tumor cells [2, 9–11]. However, little is known about the underlying mechanisms and the phenotypic advantages provided to cells by reduced miRNA expression. Interestingly, in thyroid cancer, differentiated PTC and FTC present both up- and downregulated miRNAs, whereas dedifferentiated and aggressive ATC show almost exclusively downregulated miRNAs, with various reports describing 44 downregulated miRNAs and only 6 upregulated miRNAs [12–14]. This suggests that the progression of differentiated thyroid carcinomas to aggressive ATC is characterized by changes in miRNA expression, and that miRNA downregulation might play a role in this transition. Although the mechanism by which miRNAs are underexpressed in cancer remains unknown, it could involve the RNAse III-type enzyme DICER1, which plays a fundamental role in processing miRNA precursors to mature miRNAs [1]. Generally, lower levels of DICER1 correlate with worse outcomes in lung, breast, skin, endometrial, and ovarian cancer [2]. Also, recurrent somatic mutations have been detected in metal-binding residues within the RNase IIIb domain of DICER1, decreasing the expression of some tumor-suppressive miRNAs, which likely helps to explain the selective pressures that give rise to this specific spectrum of mutations in several cancer types [2], including thyroid cancer [8, 15, 16]. Some germline mutations in DICER1 are also found in different types of inherited tumors, leading to an increased predisposition to differentiated thyroid carcinoma (PTC or FTC) [16–19]. However, the precise role that DICER1 plays in thyroid tumor progression is not clear.

Important insights into the potential role of Dicer1 in tumorigenesis have been provided by its conditional deletion in thyroid follicular cells during mouse development [20, 21]. These mice were hypothyroid at birth, and presented loss of thyroid follicular structure with downregulation of differentiation markers [20], or showed characteristics of neoplastic transformation in the thyroid tissue at adult stages [21]. These findings suggest that miRNAs are necessary to maintain thyroid tissue homeostasis and that Dicer1 could be involved in thyroid tumorigenesis.

Despite their impact on cancer biology, miRNA-based cancer therapy is still in its early stages, and almost no studies have addressed thyroid cancer. We recently described that intratumoral administration of a synthetic anti-miRNA inhibiting miR-146b blocked tumor growth of human thyroid tumor xenografts [22]. Strategies to restore global miRNA expression would be a welcome addition to the current therapeutic arsenal for thyroid cancer. In this respect, the small-molecule enoxacin, which promotes miRNA processing in a TRBP-dependent manner [23], was shown to have cancer growth inhibitory effects [24], although none of these studies were performed in thyroid cancer.

With this information, we aimed to study the function and regulation of DICER1 in thyroid cancer, and to test whether its impaired function resulted in global miRNA downregulation contributing to a more aggressive phenotype.

## Results

### The major upregulated miRNAs in thyroid cancer, including miR-146-5p, target DICER1, promoting in vitro cell proliferation, migration and invasion

**Fig. 1:**
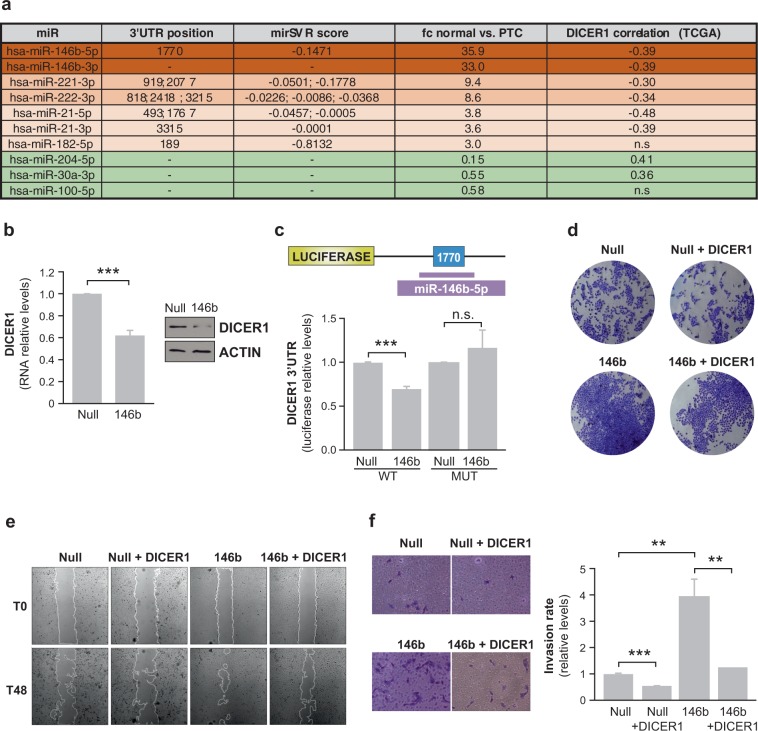
miR-146b directly targets DICER1, which in turn blocks miR-146b-induced proliferation, migration and invasion. **a** Table shows the main up- and downregulated miRNAs in thyroid cancer [25] and their predicted binding sites in the DICER 3′UTR (position and the mirSVR score for the miRs predicted by miRanda). Also shown is the fold change (FC) of normal vs PTC and the correlations between DICER1 and miRNAs using Cancer Regulome analysis in TCGA database. **b**, **c** Stable cell lines were generated from Nthy-ori cells transfected with a pEGP-Null vector (Null cells) or a pEGP-miR-146b vector (146b cells). **b** Left: relative *DICER1* expression by qPCR. Right: immunoblot of DICER1 expression (results are representative of 3 experiments). **c** Direct targeting of DICER1 3′UTR by miR-146b. Luciferase reporter activity relative to *Renilla* level was evaluated in cells 72 h after transfection of pIS1 DICER1 long UTR (WT) or DICER1 3′UTR mutated in the miR-146b binding site (MUT). **d** Representative images of crystal violet-stained cells 48 h after transfection with the DICER1 expression vector. **e** Representative images of a wound healing assay 0 and 48 h after scratching. **f** Relative quantification of the invasive capacity of cells was analyzed using Matrigel-coated Transwell assays. Left: representative images of the lower chamber (invading cells). Right: cell invasion relative to that of “Null” cells. Values represent mean ± SD (*n* = 3). **p < 0.01; ***p < 0.001; n.s. non-significant.

Recently, a specific miRNA signature of highly differentially expressed miRNAs has been established in thyroid cancer by TCGA [8], and in a cohort of patients by our laboratory [25]. Among the top overexpressed miRNAs in our study were miR-146b-5p, miR-146b-3p, miR-221-3p, miR-222-3p, miR-21-5p, miR-21-3p, and miR-182-5p. To evaluate the functional relevance of this signature for thyroid cancer outcome, we examined the downstream critical targets of these miRNAs using the in silico target screening algorithm miRanda. The results of this analysis identified *DICER1* as a putatively shared target of the main miRNAs, potentially forming a miRNA biogenesis regulatory network. Coincidently, the 3′UTR of *DICER1* contained several predicted binding sites for all of these miRNAs, with high probability mirSVR scores (Fig. 1a). By contrast, almost none of the previously described underexpressed miRNAs in thyroid cancer, such as miR-204, miR-30a, and miR-100 [25], were predicted to target *DICER1* (Fig. 1a). These findings raise the possibility that some upregulated mature miRNAs act in concert as negative feedback regulators to control *DICER1* expression in thyroid cancer, whereas the downregulated miRNAs may be indirectly affected. Analysis of TCGA database using the Cancer Regulome tool showed that the expression of the most highly upregulated miRNAs in PTC—miR-146b-5p, miR-146b-3p, miR-21-3p, miR-21-5p, miR-221-3p, and miR-222-3p—negatively correlated with *DICER1* mRNA levels (Fig. 1a). We validated this result using a focused small-scale screen by transiently transfecting each miRNA individually into the thyroid cell line Nthy-ori 3-1, finding that the protein level of DICER1 was reduced in each case (Fig. S1a).

miR-146-5b is the top overexpressed miRNA in PTC [25] with >35-fold-higher expression over normal thyroid tissue and also with a high representation in the PTC miRNome [25]. It yielded a reasonable mirSVR score for the DICER1 3′UTR (−0.1471, position nt 1770) (Fig. S1b) and there was a good negative correlation (−0.39) to DICER1 expression in TCGA dataset (Fig. S1c). Moreover, miR-146b is one of the most extensively studied miRNAs in this tumor type [25] and appears to be a prognostic factor for PTC, as it associates with aggressive clinicopathological features and poor clinical outcome [26]. We recently identified PTEN and CDH1 as miR-146b targets [22]; however, the full impact and relevance of this particular miRNA as an oncogenic player has not been explored in depth. Thus, we focused our attention on this miRNA and evaluated other downstream targets that may explain its oncogenic features.

We first corroborated that DICER1 is a bona fide target of miR-146b by analyzing its expression in normal human thyroid follicular Nthy-ori 3-1 cells stably overexpressing miR-146b (Nthy-ori-146b) or a control vector (Nthy-ori-Null). Results showed that DICER1 mRNA and protein levels were reduced in cells overexpressing miR-146b (Fig. 1b), consistent with the findings of our focused DICER1-miRNA target screen (Fig. S1a). Moreover, ectopic miR-146b expression significantly decreased the activity of a co-transfected luciferase reporter construct containing the putative wild-type miR-146b binding region in the *DICER1* 3′UTR (Fig. 1c). However, non-significant changes were observed when the 3′UTR DICER1 luciferase construct was mutated in the predicted binding site for miR-146b (Fig. 1c). Overall, these data show that miR-146b directly represses DICER1 expression by targeting its 3′UTR.

Given these results, we investigated the role of DICER1 in the aggressive traits induced by miR-146b overexpression, finding that overexpression of DICER1 cDNA partly rescued the miR-146b-induced increase in proliferation, migration, and invasion (Fig. 1d–f). The finding that miR-146b overexpression induces a global downregulation of miRNAs, including important tumor suppressor miRNAs such as miR-30a-5p, miR-30a-3p, miR-100, and miR-204 (Fig. S1d), suggests that the aggressiveness traits induced by this miRNA are likely elicited by DICER1 inhibition. Overall, these results show that the 3′UTR of DICER1 contains putative binding sites for the most highly overexpressed miRNAs (miR-21-3p, miR-21-5p, miR-221-3p) and that miR-146-5p directly targets *DICER1*, mediating the potential oncogenic effects and aggressiveness of thyroid cancer cells in vitro.

### Neutralization of miR-146b suppresses growth of established thyroid tumors in vivo and restores DICER1 expression

**Fig. 2:**
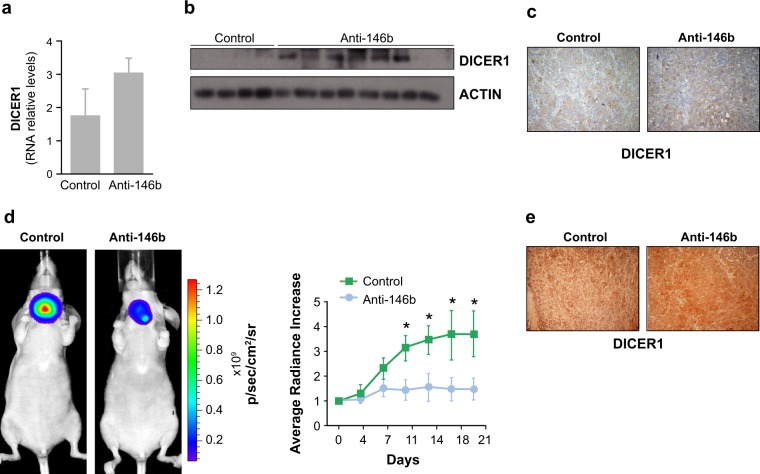
miR-146b inhibition impairs established human thyroid tumor growth. **a**–**c** Tumor samples taken from mice treated with a control or an miR-146b inhibitor (Anti-146b) administered intratumorally to tumor xenografts generated as previously described [22]. Tumors were analyzed for DICER1 expression by **a** qPCR, **b** immunoblotting, and **c** immunohistochemistry. Actin was used as a loading control. **d** Cal62-luc cells were injected into the right thyroid lobe and an orthotopic thyroid tumor was generated. Then, a synthetic miR-146b inhibitor (Anti-146b) or a negative control was administered systemically via the retro-orbital vein. Left: endpoint (day 21) bioluminescent signal of the treated tumors. Right: tumor radiance quantification at the indicated time points in mice from treatment onset with the miRNA inhibitor (blue) or the negative control (green). **e** Representative immunohistochemistry with an anti-DICER1 antibody in orthotopic tumors. Values represent mean ± SEM. *p < 0.05

We next asked if the continuous repression of DICER1 by endogenous miR-146b in aggressive thyroid cells is required for tumor growth in vivo. To test this, we first used our recently developed heterotopic xenograft model [22], in which established solid tumors of Cal62 cells were treated intratumorally with a specific anti-miR-146-5p oligonucleotide inhibitor (Anti-146b) or a control. Tumor growth was significantly blunted in the anti-miRNA-treated tumors, as we previously demonstrated [22]. We then analyzed DICER1 mRNA and protein levels in these xenograft tumors, finding that intratumoral anti-miRNA treatment rescued the levels of DICER1 (Fig. 2a–c). This suggests that restoration of DICER1 expression by intratumoral inhibition of miR-146b contributes, at least in part, to tumor growth reduction. To better assess the therapeutic potential of anti-miR-146b, we generated an orthotopic mouse thyroid tumor model. After tumor establishment (3 weeks), 13 mice were randomized into two groups and were injected intravenously with anti-miR-146b (*n* = 8) or an appropriate control (*n* = 5). Tumor volume was followed for a further two weeks. Results showed that tumor growth was significantly delayed in the anti-miRNA-treated group (Fig. 2d). Notably, DICER1 expression in the primary thyroid tumor was higher after systemic anti-miR-146b treatment (Fig. 2e). We did not observe evident adverse effect of the systemic anti-miR146b treatment in the studied parameters, as liver morphology and the levels of serum glucose were similar in both control and treated mice (Fig. S2a and b, respectively).

### DICER1 plays a critical tumor-suppressive role in thyroid cancer progression

The expression levels of DICER1 were lower in a panel of thyroid cancer cells than in the control Nthy-ori cells (Fig. S3a). To examine the effects of DICER1 on tumor-specific phenotpyes in vitro, we performed loss-of-function and gain-of-function studies using the cell lines Cal62 and TPC1, which have intermediate levels of DICER1, and with SW1736, which has low levels of DICER1 (Fig. S3a).

**Fig. 3:**
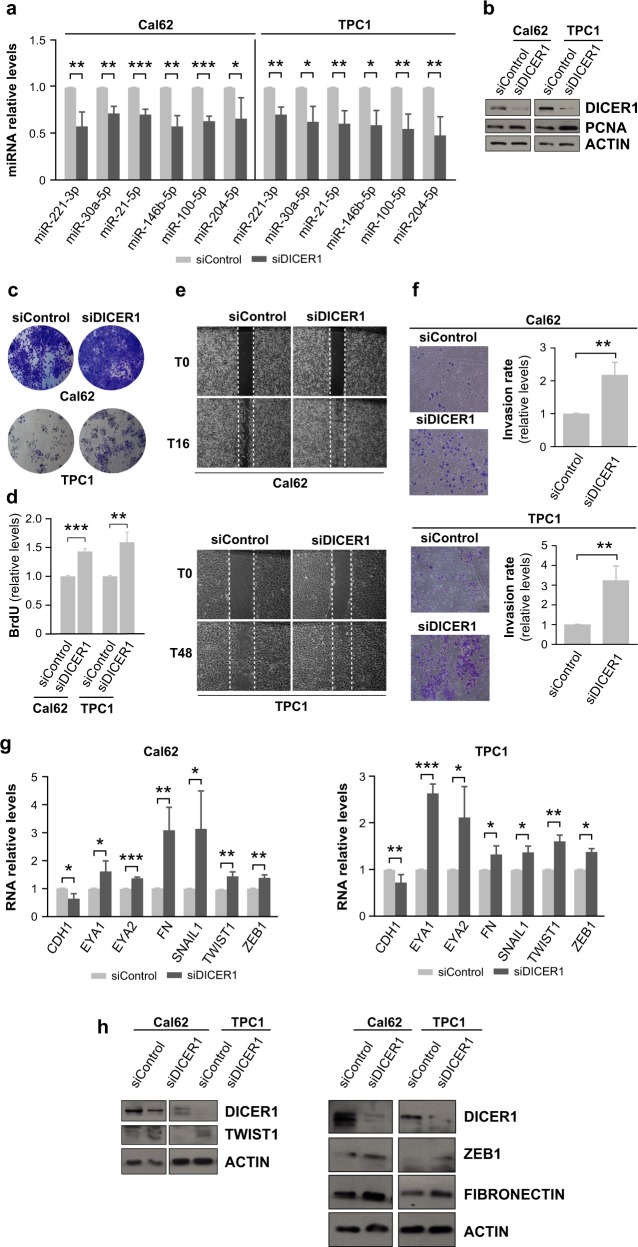
DICER1 silencing increases proliferation, migration and invasion in vitro. **a**–**f** Cal62 and TPC1 cell lines were transfected with an siRNA against DICER1 (siDICER1) or a control siRNA (siControl) and analysis was performed 48 h later. **a** Relative expression levels of miRNAs. **b** Immunoblot of DICER1 and proliferating cell nuclear antigen (PCNA). Actin was used as a loading control. **c** Representative images of crystal violet-stained cells. **d** BrdU incorporation relative to siControl-transfected cells. **e** Representative images from a wound healing assay at 0 and 16 h (Cal62, left) or 0 and 48 h (TPC1, right) after scratching. **f** Quantification of the invasion rates. Left: representative images of the lower chamber (invading cells). Right: cell invasion rates relative to siControl cells. **g**, **h** Cells were transfected with an siRNA against DICER1 (siDICER1) or a control siRNA (siControl). **g** Relative mRNA levels assayed by qRT-PCR of EMT genes *CDH1*, *EYA1*, *EYA2*, *FN*, *PAI1*, *SNAIL1*, *TWIST1*, and *ZEB1* 72 h after siRNA transfection with RNAiMAX Lipofectamine. **h** Immunoblot for DICER1 and TWIST1 (left) and DICER1, ZEB1, and fibronectin (right) 48 h after siRNA transfection. Actin was used as a loading control. Values represent mean ± SD (*n* = 3). *p < 0.05; **p < 0.01; ***p < 0.001

Silencing of DICER1 in Cal62 and TPC1 cells resulted in a significant decrease in the expression of several mature miRNAs (miR-221-3p, miR-30a-5p, miR-21-5p, miR-146b-5p, miR-100-5p, and miR-204-5p) (Fig. 3a). As expected, the expression levels of pre-miRNAs were essentially stable (Fig. S3b). Moreover, proliferation (by PCNA immunoblotting, cell viability, and DNA synthesis) (Fig. 3b–d), and also migration and invasion (Fig. 3e, f), were all higher in DICER1-silenced cells than in controls. We also observed that the expression of protein markers involved in epithelial-mesenchymal transition (EMT) was altered in DICER1-silenced cells at 72 h post-transfection. Whereas DICER1 silencing significantly decreased the mRNA levels of the epithelial protein E-cadherin (CDH1), it increased the expression of several mesenchymal genes/proteins, including EYA1, EYA2, fibronectin, SNAIL1, TWIST1, and ZEB1, in both cell lines (Fig. 3g, h). These data demonstrate that DICER1 is a critical player in thyroid tumor progression, confirming that DICER1 knockdown copies the pro-metastatic phenotypes observed by exogenous miR-146b overexpression.

In contrast to the loss-of-function studies, SW1736 cells ectopically overexpressing DICER1 showed an expected induction of several DICER1-dependent mature miRNAs (Fig. S4a), and proliferation, migration, and invasion were all significantly decreased together with the corresponding changes in the EMT markers (Fig. S4b–g). Overall, these data unequivocally establish a role of DICER1 as a tumor suppressor in thyroid follicular cells, confirming previous data demonstrating this tumor suppressor effect in other tumor types [2, 9–11].

### Association between DICER1 expression and worse clinical outcome in thyroid cancer patients

**Fig. 4:**
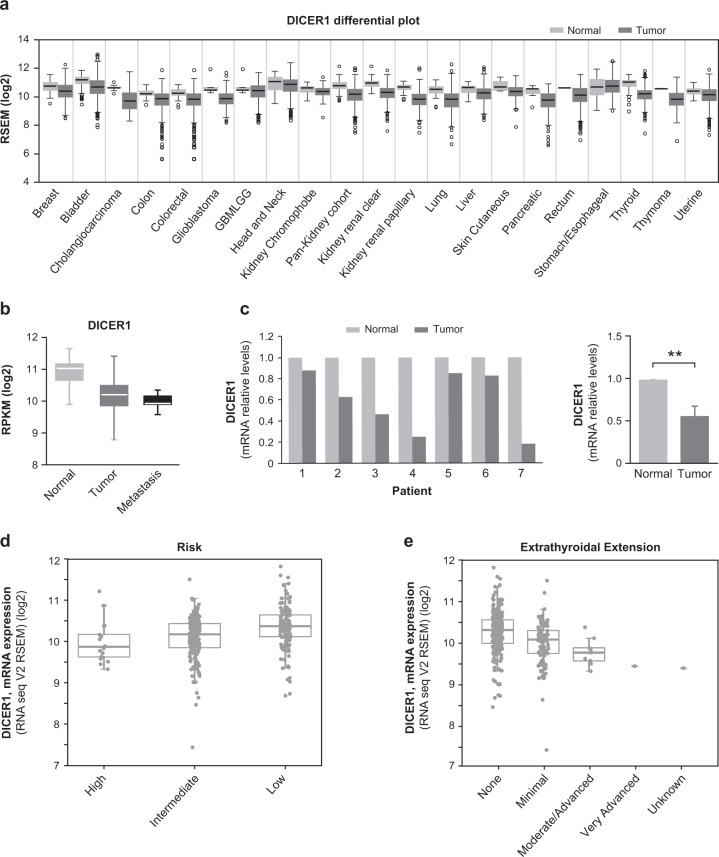
DICER1 is downregulated in human PTC tumors. **a** DICER1 mRNA expression levels in the indicated cancer types obtained by Firebrowse analysis of the TCGA database. **b** Box plot of DICER1 mRNA expression levels in thyroid normal tissue, PTC, and metastases: data were obtained from the TCGA database (normal vs tumor *p*-value < 0.001) (Number of samples: 59 control, 501 primary solid tumors and 8 metastasis). **c** Left: relative DICER1 mRNA levels in 7 PTC patients (contralateral and normal thyroid tissue). Right: total average of DICER1 mRNA relative levels. **d**, **e** Correlation between DICER1 mRNA levels in high, intermediate or low risk (*p*-value < 0.001, adjusted *p*-value < 0.01) (**d**), or extrathyroid extension (*p*-value > 0.001, adjusted *p*-value = 0.3) (**e**), by analyzing the cBioPortal, where the TCGA datasets are deposited. Values represent mean ± SEM. **p < 0.01

The clinical relevance of DICER1 repression in thyroid cancer was analyzed in tumor samples and normal tissues using data acquired from TCGA FireBrowse portal and the Morpheus tool. We observed that DICER1 levels were decreased in several cancer types (Fig. 4a), and thyroid cancer showed the third greatest change in DICER1 expression overall when compared with normal tissue (Fig. 4b) (normal tissue *n* = 59, tumor samples *n* = 501). Of note, thyroid metastases (*n* = 8) exhibited even lower levels of *DICER1* than primary tumor samples or normal tissue (Fig. 4b), pointing to a role for DICER1 downregulation in tumor progression. To extend these observations, we surveyed DICER1 mRNA levels in an independent paired cohort of 7 PTC patients (clinical characteristics are summarized in Table S1), finding that levels were lower in tumor samples than in contralateral normal thyroid tissue in most patients (Fig. 4c, left). Globally, we observed a significant decrease in DICER1 mRNA levels of ~46% in these patients (Fig. 4c, right).

To further explore the implications of DICER1 dysregulation in thyroid cancer, we sought to study its correlation with aggressive clinical features. TCGA data from the public databases cBioportal and Cancer Regulome indicated that DICER1 levels were inversely correlated with the risk of recurrence (Fig. 4d) and with extrathyroidal extension (Fig. 4e). Moreover, DICER1 was more downregulated in tumors classified within the miRNA clusters 5 and 6, described in TCGA as the less-differentiated tumors with high risk of recurrence (Figure S5a) [3, 8]. Overall, these data strongly suggest an important role for DICER1 in thyroid tumor progression.

Because the maturation of most miRNAs is blocked in the absence of DICER1 [27], we analyzed the global expression of the miRNAs differentially expressed in thyroid cancer. Through the analysis of TCGA data, we found 93 miRNAs differentially expressed between samples of PTC and normal tissue: whereas 20 miRNAs were upregulated, as many as 73 were downregulated approximately ~3.5-fold or more (false discovery rate [FDR] < 0.01 and fold change [FC] > 2 for upregulated miRNAs and FC < 0.75 for downregulated miRNAs) (Fig. S5b). The higher number of downregulated miRNAs relative to upregulated miRNAs in thyroid tumors suggests an important role of potential tumor-suppressive miRNAs in this cancer type, and are in line with the findings of DICER1 downregulation.

### Individual downregulated miRNAs rescue DICER1-silencing effects

Our data thus far clearly establish a direct role of DICER1 repression by miR-146-5p and potentially other miRNAs in PTC. Given its tumor suppressor effect, we next sought to address further downstream DICER1 effector miRNAs relevant to this phenotype. We and others recently profiled repressed miRNAs in PTC with potential tumor suppressor functions [8, 25], which highlighted miR-30a and miR-100. These miRNAs are reported to be essential for EMT and the mesenchymal phenotype by, targeting lysyl oxidase and promoting SNAIL2 expression [13, 28, 29], and have been described as tumor suppressors in other cancer types. We confirmed the tumor suppressor functions of miR-30a and miR-100 in Cal62 cells, as their individual transfection decreased proliferation (Fig. S6a, b) and invasion (Fig. S6c).

**Fig. 5:**
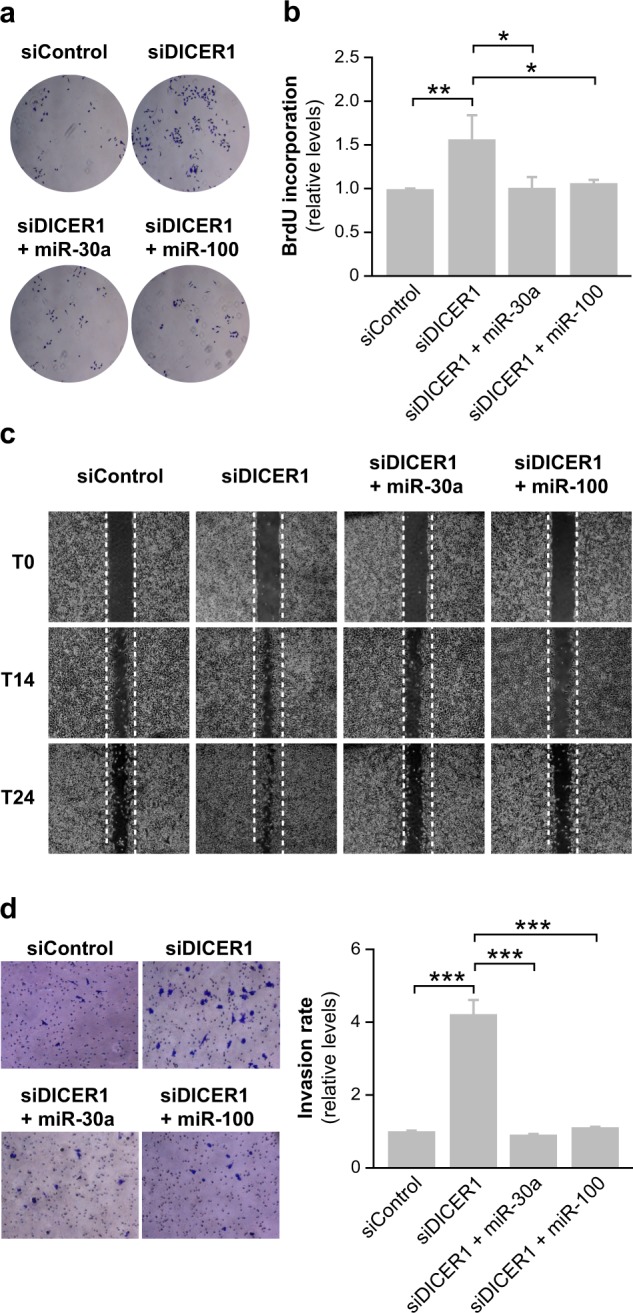
Downregulated miR-30a and miR-100 in thyroid cancer block DICER1-silencing-induced effects. Cal62 cells were silenced for DICER1 (siDICER1) and co-transfected with miR-30a or miR-100 plasmids. Control cells were transfected with siControl and the empty DICER1 vector. **a** Representative images of cells transfected with siControl, siDICER1, siDICER1 + miR-30a, and siDICER + miR-100 at 48 h post-transfection and stained with crystal violet. **b** BrdU incorporation. **c** Representative images from a wound healing assay 0, 14, and 24 h after scratching in the above-mentioned conditions. **d** Quantification of invasion capacity was analyzed in Matrigel-coated Transwells. Left: representative images of the lower chamber (invading cells). Right: cell invasion relative to siControl cells. Values represent mean ± SD (*n* = 3). *p < 0.05; **p < 0.01; ***p < 0.001

To assess whether the effects of DICER1 silencing are directly attributed to these repressed miRNAs, we restored miR-30a and miR-100 expression in DICER1-silenced cells and monitored the resulting phenotype. Indeed, transfection of miR-30a or miR-100 reversed the DICER1-silenced phenotypes, and decreased proliferation (Fig. 5a, b), migration (Fig. 5c) and invasion (Fig. 5d). These results indicate that inhibition of miRNAs with tumor suppressor functions, such as the DICER1 effectors miR-30a or miR-100, is critical for the proliferative and motile properties of malignant tumoral thyroid cells.

### Induction of miRNA processing with enoxacin decreases cell aggressiveness in vitro and tumor growth in vivo

**Fig. 6:**
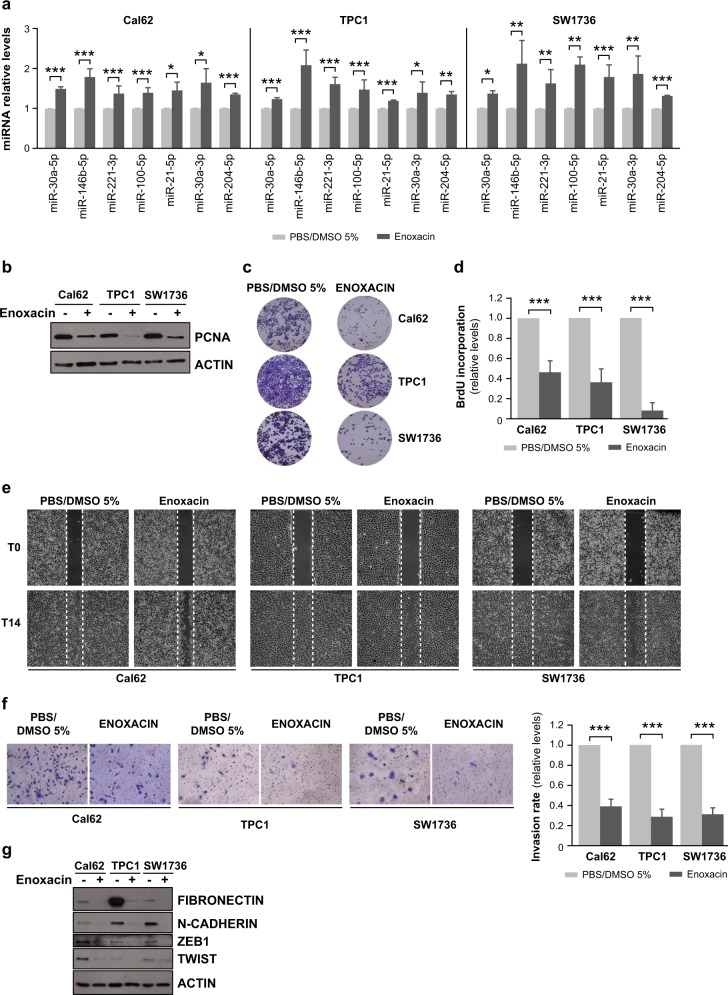
Enoxacin induces miRNA expression and decreases cell proliferation, migration and invasion in vitro. Cal62, TPC1, and SW1736 cell lines where treated with enoxacin or PBS + 5% DMSO (control) for 5 days. **a** Relative expression levels of miR-30a-5p, miR-146b-5p, miR-221-3p, miR-100-5p, miR-21-5p, miR-30a-3p, and miR-204-5p in cells. **b** Immunoblot of proliferating cell nuclear antigen (PCNA) and actin, as a loading control. **c** Representative images of cells treated as described and stained with crystal violet. **d** BrdU incorporation of cells treated with enoxacin relative to control-treated cells. **e** Representative images from a wound healing assay 0 and 14 h after scratching. **f** Quantification of invasion rates. Left: representative images of the lower chamber (invading cells). Right: cell invasion rates relative to control-treated cells. **g** Immunoblot for fibronectin, N-cadherin, ZEB1, and TWIST1. Values represent mean ± SD (*n* = 3). *p < 0.05; **p < 0.01; ***p < 0.001

Although many studies have investigated the impact of miRNAs on cancer biology, miRNA-based cancer therapy is still in its early stages and is mostly limited to targeting a single miRNA [30, 31]. However, because global downregulation of miRNA expression and corresponding DICER1 repression appears to play a role in thyroid cancer, restoration of their levels or enhancing miRNA biogenesis might represent an attractive approach in cancer therapy. Against this background, we treated Cal62, TPC1 and SW1736 cells with the small-molecule enoxacin, which enhances miRNA maturation by binding to TRBP. Consistent with its activity against other types of tumors [24], the enhancement of DICER1 complex enzymatic activity by enoxacin resulted in an increase in the expression of all tested mature miRNAs, including tumor suppressor miRNAs (Fig. 6a). Also, enoxacin markedly inhibited proliferation, migration, and invasion in Cal62, TPC1, and SW1736 cells (Fig. 6b–f), and decreased the expression of EMT markers (Fig. 6g). By contrast, enoxacin had less effect on non-transformed thyroid Nthy-ori 3-1 cells, as shown by a slight decrease in proliferation (Fig. S7a, b), but no changes in invasion (Fig. S7c).

**Fig. 7:**
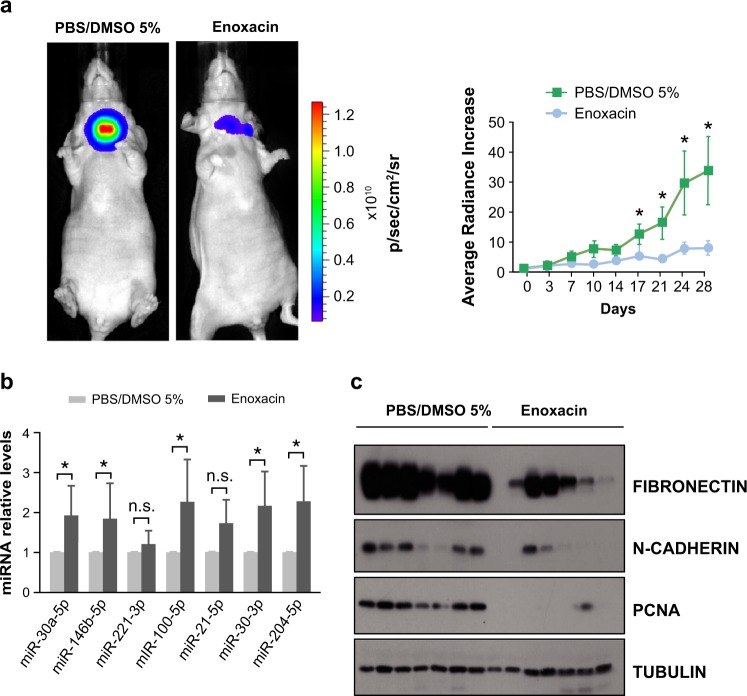
Enoxacin impairs established human orthotopic thyroid tumor growth and decreases the expression of EMT-related genes in vivo. **a**–**c** Cal62-luc cells were injected into the right thyroid lobe. After the orthotopic model was established, enoxacin or vehicle was administered intraperitoneally. **a** Left: the image shows the endpoint (day 28) bioluminescent signal of the tumors imaged with the IVIS-Lumina II Imaging System. Right: tumor radiance quantification at the indicated time points in mice from treatment onset with enoxacin (blue) or vehicle (green). **b** RNA was obtained from the tumors and miRNA expression levels were determined. Shown are the relative intratumoral levels of miR-30a-5p, miR-146b-5p, miR-221-3p, mIR-100-5p, miR-21-5p, miR-30-5p, and miR-204-5p. **c** Immunoblot of intratumoral expression of fibronectin, N-cadherin, and PCNA in enoxacin- or vehicle-treated mice. Values represent mean ± SEM. *p < 0.05; n.s. non-significant

Finally, to translate these in vitro findings to an in vivo thyroid cancer model, we tested enoxacin in the orthotopic mouse model of human thyroid cancer. After establishing the tumors, 15 mice were randomized into two groups: a test group (*n* = 8) treated daily by intraperitoneal injection of enoxacin (15 mg/kg) over 28 days, and a vehicle control group (*n* = 7) treated with 5% DMSO in phosphate buffered saline (PBS). We found that enoxacin significantly diminished the radiance bioluminescence increase (Fig. 7a left) and decreased tumor growth (Fig. 7a right). We confirmed the upregulated expression of critical miRNAs in tumors from enoxacin-treated mice (Fig. 7b), and the downregulation of the EMT markers fibronectin and N-cadherin and the proliferation marker PCNA (Fig. 7c). The systemic treatment with the drug did not produce adverse effects in mice, as demonstrated by the similar levels of glucose in both groups analyzed (Fig. S2c)

Overall, these data suggest that enoxacin enhances miRNA production and, consequently, re-establishes DICER1-miRNA processing, eliciting its antitumor effect.

## Discussion

**Fig. 8:**
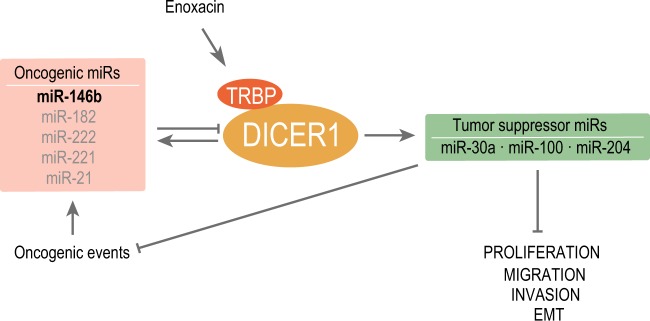
Summary of established DICER1-miRNA regulatory network in thyroid cancer. Overexpressed (oncogenic) miRNAs in thyroid cancer target DICER1. miR-146b (bold) directly binds to DICER1 3′UTR; other miRNAs (grey) have a predicted biding site in DICER1 3′UTR. Also, DICER1 upregulates tumor suppressor miRNAs, which in turn inhibit proliferation, migration, invasion, and EMT. This effect can be phenocopied when increasing the DICER1 complex activity with the small-molecule enoxacin. The arrows denote induction and the truncated lines repression

We provide evidence that thyroid cancer cells use global downregulation of a miRNA sub-network to enhance disease aggressiveness, including the induction of proliferation and metastatic capacity. Global downregulation of miRNA maturation has been described in many tumor types [2, 9–11, 32] including thyroid cancer, where it has been suggested that distinct miRNA expression patterns can distinguish ATC from FTC or PTC [14]. Our results give direct support for and extend this concept by identifying a mechanism by which miRNA down-modulation leads to thyroid tumor progression. We show that the most overexpressed miRNA in thyroid cancer, miR-146b, targets DICER1, inhibiting its expression. Furthermore, the 3′UTR of DICER1 also contains putative binding sites for other upregulated miRNAs (miR21, -222, -221, -182) in thyroid cancer (Fig. 8). These results are reinforced by our exhaustive analysis of TCGA data [8]. We focused our attention on miR-146b, as we have recently characterized its oncogenic activity in thyroid cancer [22]. The miR-146b-DICER1 interaction revealed in this work has profound consequences for the induction of an aggressive phenotype, as ectopic DICER1 expression rescues the aggressive phenotype induced by miR-146b. Since miR-146b is both produced by and regulates DICER1, this mutual feedback relationship allows for a low level of DICER1 in thyroid cancer but not its complete loss, and thus sufficient DICER1 levels are maintained for cell survival and growth.

Repression of DICER1 is not the only oncogenic action of miR-146b, as we have demonstrated that it also represses the tumor suppressor PTEN, leading to an activation of the oncogenic PI3K/AKT pathway [22]. We believe that activation of PI3K/AKT together with the global downregulation of miRNAs could contribute to a more aggressive tumor phenotype. In our in vivo studies, anti-miR-146b treatment suppressed tumor growth and reversed DICER1 expression. These findings have implications for the treatment of thyroid cancer suggesting that modulation of miR-146b by RNA-based therapeutics could be clinically useful.

Conceptually, some of the present data may seem paradoxical, as DICER1 is downregulated in thyroid cancer, but some miRNAs are upregulated. However, it is known that, among other phenomena, the activation of oncogenes causes upregulation of specific miRNAs [33]. For example, conditional activation of BRAFV600E and RET/PTC3 in normal thyroid cells induces the expression of miR-146b [34], which can explain the co-existence of a general miRNA downregulation in tumors with low DICER1 levels and the upregulation of miR-146b. In addition, other oncogenic pathways in thyroid cancer have been described to increase miR-146b levels such as NF-κB [35, 36] or PI3K/AKT pathways [22]. Another possible explanation for miRNA upregulation could be gene demethylation. Consistent with this is the result of our analysis of TCGA database, showing that miR-146b is demethylated in thyroid cancer and its metastasis (Fig. S8), whereas downregulated miRNAs have almost no change in methylation levels (not shown).

The analysis of TGCA data showed an association between low DICER1 expression and thyroid metastasis, high risk, and extrathyroidal extensions. Indeed, we confirmed low DICER1 mRNA levels in a panel of seven PTC thyroid tumors. Taken together, these data suggest that, similar to other tumor types [2, 37, 38], low DICER1 levels in thyroid cancer are associated with advanced tumor stage and poor clinical outcome [39]. Moreover, the present work provides functional evidence that DICER1 acts as a strong tumor suppressor in thyroid cancer, confirming data in other tumor types.

In loss- or gain-of-function experiments, we observed that DICER1 suppress cell aggressiveness (Fig. 8). Our data are in contrast to a report showing that DICER1 overexpression positively regulates thyroid cell proliferation [40], but are in agreement with the observation that *Dicer1* knockout in embryonic thyroid results in a marked increase of proliferation [21]. In addition, the extensive analysis of TCGA data and our work herein strongly support that DICER1 acts as a tumor suppressor and not an oncogene.

We have demonstrated that global miRNA downregulation induced by DICER1 silencing promotes thyroid cell aggressiveness. Notably, thyroid cancer progression leads to fewer upregulated and more downregulated miRNAs [12, 13]. This observation is consistent with the hypothesis that the biogenesis machinery breaks down as the tumor moves towards a more aggressive stage. That an aggressive tumoral behavior becomes manifest after the general downregulation of miRNAs points to an important contribution of this process to the aggressive phenotype, including the downregulation of tumor suppressor miRNAs. This is supported by our data in DICER1-silenced cells where the expression of tumor suppressors miR-30a and miR-100 recover the normal thyroid phenotype.

It is important to find strategies to restore global miRNA expression as a potential therapy in thyroid tumors. Regarding the restoration of miRNAs with tumor suppressor roles, few examples have been reported, but it is reasonable to believe that restoring global miRNA levels could have a therapeutic effect. Along this line, we used the small-molecule enoxacin to enhance the production of miRNAs, observing that this molecule reduces cell aggressiveness in vitro and tumor growth in vivo without signs of toxicity in animals [24]. The finding that enoxacin treatment of non-transformed thyroid cells had less effect than in tumoral cells suggests that this inductor of miRNA biogenesis has a more specific action in tumoral thyroid cells. In addition, we provide evidence that activating miRNA biogenesis is a potentially important step towards the application of miRNA-based therapy for the treatment of thyroid cancer. This therapy is promising in other pathologies [41].

In summary, our work reveals some key aspects on the regulation and function of DICER1 in thyroid cancer. Paradoxically, DICER1 is downregulated in thyroid cancer through the upregulation of targeting miRNAs, particularly miR-146b. DICER1 repression functionally leads to global downregulation of the miRNA network, inducing increased malignancy. Our data may have important clinical implications as DICER1 may convey prognostic information and, more importantly, point to a potential therapeutic approach based on the restoration of global miRNA levels via the DICER1 pathway or using DICER1-targeting miRNA inhibitors in patients with thyroid and other cancers.

## Material and methods

### Bioinformatic predictions

The TCGA database was queried to assess correlations between mRNA and miRNA levels and clinical features. Firebrowse (http://firebrowse.org) was used to analyze *DICER1* mRNA levels in different tumor types. The cBioPortal (http://www.cbioportal.org) and the Cancer Regulome Explorer (http://explorer.cancerregulome.org) data portals were used to obtain the correlations using the thyroid carcinoma dataset (THCA). The miRanda algorithm (http://www.miRNAs.org) was used to predict hypothetical associations between miRNAss and mRNAs.

### Patients

Samples of PTC tumors and contralateral normal thyroid tissue from the same patients (*n* = 7) were collected at the Biobank of the Hospital Universitario La Paz (Madrid, Spain). A summary of the main clinical characteristics of the patients is included in Table SI. Written informed consent was obtained from all the patients in accordance with the protocols approved by the local ethics committee.

### RNA quantification

RNA was extracted using TRIzol (Invitrogen). RT-PCR and qRT-PCR were performed as described in Supplementary Materials and Methods. The primer sequences used in this study are listed in Table SII.

### Cell culture and transfection

The human thyroid cancer cell lines of PTC (BCPAP and TPC1) and ATC (Ocut2, Ktc2, Cal62, T235, Hth83, Hth74, and SW1736) were cultured as described [42]. Rat PCC13 cells [43] and the human primary thyroid follicular cell line Nthy-ori 3-1 [22], derived from normal thyroid tissue, were cultured as described. Stable cell lines, transfections, and Luciferase activity were performed as described in Supplementary Materials and Methods.

### Plasmids, constructs, and siRNA

The *DICER1* expression vector was kindly provided by Dr Richard Gregory (Boston Children’s Hospital, Boston) [44]. The pCMV2-miR30a vector was a gift from Dr. Bryan Cullen (Addgene plasmid #20670) [45] and the pIS-DICER1 long UTR was a gift from Dr David Bartel (Addgene plasmid #21649) [46]. This construct was mutated in the miR-146b binding site by site-directed mutagenesis as described in Supplementary Material and Methods.

The luciferase and GFP-expressing vector, CMV-Firefly luc-IRES-EGFP, was constructed by Dr. J. Bravo (IQAC-CSIC), and the Cal62 human tumoral thyroid cell line stably expressing this vector (Cal62-Luc) was generated by Dr. Eugenia Mato (IB, Sant Pau).

The pre-miR-146b construct was previously cloned into a pEGP expression vector (Cell Biolabs) [22]. The pre-miR-100 miRNAs were amplified from genomic DNA using the follow primers: 5′-TCGAACGCGTTCCACCTCAGCCCCCTTTTC-3′ (forward) and 5′-TCGAGAGCTCTGGGACGAAGTCCTTTCCATTT-3′ (reverese) and cloned into the pEGP-miR expression vector. DICER1 siRNA was purchased from Thermo Fisher (Silencer® Select Pre-Designed siRNA Dicer1, s23754).

### Protein extraction, western blotting, and immunohistochemistry

Cells were lysed and proteins extracted as described [22]. Western blotting and immunohistochemistry procedures are detailed in Supplementary Materials and Methods.

### Cell proliferation assays

Proliferation was determined by proliferating cell nuclear antigen (PCNA) expression, crystal violet staining and bromodeoxyuridine (BrdU) incorporation, and measured as described [22, 42]. Full details are in Supplementary Materials and Methods.

### Migration and invasion assays

Wound healing and cell invasion assays were performed as described [22], and as detailed in Supplementary Materials and Methods.

### Enoxacin treatment in vitro

To stimulate miRNA expression, cells were treated with enoxacin, sodium salt (EMD Millipore Corp.; cat. no. 557305) at 40 µg/mL diluted in PBS with 5% DMSO, for 5 days [24].

### In vivo studies

Animal experimentation was performed in compliance with the European Community Law (86/609/EEC) and the Spanish law (R.D. 1201/2005), with the approval of the ethics committee of the Consejo Superior de Investigaciones Científicas (CSIC, Spain).

Xenograft models have been extensively described previously in our previous study [22]. Orthotopic implantation was performed as described in Supplementary Materials and Methods.

### Statistical analysis

Results are expressed as the mean ± SD of at least three different experiments performed in triplicate. Results from the in vivo studies and patient analysis are expressed as the mean ± SEM. Statistical significance was determined by Student’s *t*-test analysis (two-tailed) and differences were considered significant at a *P*-value < 0.05.

## Supplementary information

Suppl. Material and Methods

Figure S1

Figure S2

Figure S3

Figure S4

Figure S5

Figure S6

Figure S7

Figure S8

Suppl. tables

Suppl. Figure Legends

## References

[1] Gregory RI, Shiekhattar R (2005). MicroRNA biogenesis and cancer. Cancer Res.

[2] Lin S, Gregory RI (2015). MicroRNA biogenesis pathways in cancer. Nat Rev Cancer.

[3] Riesco-Eizaguirre G, Santisteban P (2016). Endocrine tumours: advances in the molecular pathogenesis of thyroid cancer: lessons from the cancer genome. Eur J Endocrinol.

[4] Pallante P, Battista S, Pierantoni GM, Fusco A (2014). Deregulation of microRNA expression in thyroid neoplasias. Nat Rev Endocrinol.

[5] Lim H, Devesa SS, Sosa JA, Check D, Kitahara CM (2017). Trends in thyroid cancer incidence and mortality in the United States, 1974-2013. Jama.

[6] Haugen BR, Sherman SI (2013). Evolving approaches to patients with advanced differentiated thyroid cancer. Endocr Rev.

[7] Fuziwara CS, Kimura ET (2017). MicroRNAs in thyroid development, function and tumorigenesis. Mol Cell Endocrinol.

[8] Cancer Genome Atlas Research Network. Integrated genomic characterization of papillary thyroid carcinoma. Cell. 2014;159:676–90. 10.1016/j.cell.2014.09.050 PMC4243044 25417114

[9] Lu J, Getz G, Miska EA, Alvarez-Saavedra E, Lamb J, Peck D (2005). MicroRNA expression profiles classify human cancers. Nature.

[10] Thomson JM, Newman M, Parker JS, Morin-Kensicki EM, Wright T, Hammond SM (2006). Extensive post-transcriptional regulation of microRNAs and its implications for cancer. Genes Dev.

[11] Martello G, Rosato A, Ferrari F, Manfrin A, Cordenonsi M, Dupont S (2010). A MicroRNA targeting dicer for metastasis control. Cell.

[12] Saiselet M, Pita JM, Augenlicht A, Dom G, Tarabichi M, Fimereli D (2016). miRNA expression and function in thyroid carcinomas: a comparative and critical analysis and a model for other cancers. Oncotarget.

[13] Hebrant A, Floor S, Saiselet M, Antoniou A, Desbuleux A, Snyers B (2014). miRNA expression in anaplastic thyroid carcinomas. PLoS ONE.

[14] Braun J, Hoang-Vu C, Dralle H, Huttelmaier S (2010). Downregulation of microRNAs directs the EMT and invasive potential of anaplastic thyroid carcinomas. Oncogene.

[15] Khan NE, Bauer AJ, Schultz KAP, Doros L, Decastro RM, Ling A (2017). Quantification of thyroid cancer and multinodular goiter risk in the DICER1 syndrome: a family-based cohort study. J Clin Endocrinol Metab.

[16] Rutter MM, Jha P, Schultz KA, Sheil A, Harris AK, Bauer AJ (2016). DICER1 mutations and differentiated thyroid carcinoma: evidence of a direct association. J Clin Endocrinol Metab.

[17] Oue T, Inoue M, Kubota A, Kuwae Y, Kawa K (2008). Pediatric thyroid cancer arising after treatment for pleuropulmonary blastoma. Pediatr Blood Cancer.

[18] Rome A, Gentet JC, Coze C, Andre N (2008). Pediatric thyroid cancer arising as a fourth cancer in a child with pleuropulmonary blastoma. Pediatr Blood Cancer.

[19] de Kock L, Sabbaghian N, Soglio DB, Guillerman RP, Park BK, Chami R (2014). Exploring the association Between DICER1 mutations and differentiated thyroid carcinoma. J Clin Endocrinol Metab.

[20] Frezzetti D, Reale C, Cali G, Nitsch L, Fagman H, Nilsson O (2011). The microRNA-processing enzyme Dicer is essential for thyroid function. PLoS ONE.

[21] Rodriguez W, Jin L, Janssens V, Pierreux C, Hick AC, Urizar E (2012). Deletion of the RNaseIII enzyme dicer in thyroid follicular cells causes hypothyroidism with signs of neoplastic alterations. PLoS ONE.

[22] Ramirez-Moya J, Wert-Lamas L, Santisteban P (2018). MicroRNA-146b promotes PI3K/AKT pathway hyperactivation and thyroid cancer progression by targeting PTEN. Oncogene.

[23] Shan G, Li Y, Zhang J, Li W, Szulwach KE, Duan R (2008). A small molecule enhances RNA interference and promotes microRNA processing. Nat Biotechnol.

[24] Melo S, Villanueva A, Moutinho C, Davalos V, Spizzo R, Ivan C (2011). Small molecule enoxacin is a cancer-specific growth inhibitor that acts by enhancing TAR RNA-binding protein 2-mediated microRNA processing. Proc Natl Acad Sci USA.

[25] Riesco-Eizaguirre G, Wert-Lamas L, Perales-Paton J, Sastre-Perona A, Fernandez LP, Santisteban P (2015). The miR-146b-3p/PAX8/NIS regulatory circuit modulates the differentiation phenotype and function of thyroid cells during carcinogenesis. Cancer Res.

[26] Chou CK, Yang KD, Chou FF, Huang CC, Lan YW, Lee YF (2013). Prognostic implications of miR-146b expression and its functional role in papillary thyroid carcinoma. J Clin Endocrinol Metab.

[27] Bernstein E, Kim SY, Carmell MA, Murchison EP, Alcorn H, Li MZ (2003). Dicer is essential for mouse development. Nat Genet.

[28] Boufraqech M, Nilubol N, Zhang L, Gara SK, Sadowski SM, Mehta A (2015). miR30a inhibits LOX expression and anaplastic thyroid cancer progression. Cancer Res.

[29] Lima CR, Gomes CC, Santos MF (2017). Role of microRNAs in endocrine cancer metastasis. Mol Cell Endocrinol.

[30] Xing M (2013). Molecular pathogenesis and mechanisms of thyroid cancer. Nat Rev Cancer.

[31] Adams BD, Parsons C, Walker L, Zhang WC, Slack FJ (2017). Targeting noncoding RNAs in disease. J Clin Invest.

[32] Ozen M, Creighton CJ, Ozdemir M, Ittmann M (2008). Widespread deregulation of microRNA expression in human prostate cancer. Oncogene.

[33] Kumar MS, Lu J, Mercer KL, Golub TR, Jacks T (2007). Impaired microRNA processing enhances cellular transformation and tumorigenesis. Nat Genet.

[34] Geraldo MV, Yamashita AS, Kimura ET (2012). MicroRNA miR-146b-5p regulates signal transduction of TGF-beta by repressing SMAD4 in thyroid cancer. Oncogene.

[35] Hammond SM (2006). MicroRNAs as oncogenes. Curr Opin Genet Dev.

[36] Pacifico F, Leonardi A (2010). Role of NF-kappaB in thyroid cancer. Mol Cell Endocrinol.

[37] Su X, Chakravarti D, Cho MS, Liu L, Gi YJ, Lin YL (2010). TAp63 suppresses metastasis through coordinate regulation of Dicer and miRNAs. Nature.

[38] Yan M, Huang HY, Wang T, Wan Y, Cui SD, Liu ZZ (2012). Dysregulated expression of dicer and drosha in breast cancer. Pathol Oncol Res.

[39] Erler P, Keutgen XM, Crowley MJ, Zetoune T, Kundel A, Kleiman D (2014). Dicer expression and microRNA dysregulation associate with aggressive features in thyroid cancer. Surgery.

[40] Penha RCC, Sepe R, De Martino M, Esposito F, Pellecchia S, Raia M (2017). Role of Dicer1 in thyroid cell proliferation and differentiation. Cell Cycle.

[41] Emde A, Eitan C, Liou LL, Libby RT, Rivkin N, Magen I (2015). Dysregulated miRNA biogenesis downstream of cellular stress and ALS-causing mutations: a new mechanism for ALS. Embo J.

[42] Sastre-Perona A, Riesco-Eizaguirre G, Zaballos MA, Santisteban P (2016). beta-catenin signaling is required for RAS-driven thyroid cancer through PI3K activation. Oncotarget.

[43] Fusco A, Berlingieri MT, Di Fiore PP, Portella G, Grieco M, Vecchio G (1987). One- and two-step transformations of rat thyroid epithelial cells by retroviral oncogenes. Mol Cell Biol.

[44] Chendrimada TP, Gregory RI, Kumaraswamy E, Norman J, Cooch N, Nishikura K (2005). TRBP recruits the Dicer complex to Ago2 for microRNA processing and gene silencing. Nature.

[45] Zeng Y, Wagner EJ, Cullen BR (2002). Both natural and designed micro RNAs can inhibit the expression of cognate mRNAs when expressed in human cells. Mol Cell.

[46] Mayr C, Bartel DP (2009). Widespread shortening of 3′UTRs by alternative cleavage and polyadenylation activates oncogenes in cancer cells. Cell.

