# Supplementary material for: Impaired microRNA processing by DICER1 downregulation endows thyroid cancer with increased aggressiveness
Source: Oncogene. 2019 Apr 9;38(27):5486–99. doi: 10.1038/s41388-019-0804-8 (PMC6755984; doi:10.1038/s41388-019-0804-8)
Supplement: Supplementary file 1 — Suppl. Material and Methods [file 41388_2019_804_MOESM1_ESM.docx]

# ONC-2018-01823-RR: Ramírez-Moya et al.

**Impaired miRNA processing by DICER1 downregulation endows thyroid cancer with increased aggressiveness**

**Supplementary Material and Methods**

**RNA extraction and real-time quantitative RT-PCR analysis.** Equal amounts of RNA were added to a reverse-transcriptase (RT) reaction mix (M-MLV Reverse Transcriptase Kit; Promega Corporation). RT-PCR and quantitative (q)RT-PCR assays for mature miRNAs were performed using the NCode™ miRNA First-Strand cDNA Synthesis and the qRT-PCR Kit (Invitrogen). All primers were purchased from Sigma- Aldrich and are described in Supplementary Table II.

**Cell culture.** Stable clones of the non-transformed human thyroid cell line Nthy-ori 3-1 overexpressing miR-146b (Ramírez- Moya et al. Oncogene; 37: 3369–3383, 2018) were used. All cell lines used in this work were tested for mycoplasma contamination and authenticated every 6 months by short tandem repeat profiles using the Applied Biosystems Identifier kit in the Genomic Facility at the Institute of Biomedical Research (IIBm; Madrid, Spain). Transfections were performed using Lipofectamine 2000 or RNAiMAX Lipofectamine (Invitrogen).

**Site-directed mutagenesis.** Site-directed mutagenesis was performed to disrupt the miR-146b binding site mapped at -1770 of DICER1 3’UTR. The QuikChange II Site-Directed Mutagenesis Kit (Agilent Technologies Inc., Santa Clara, CA) was used according to the manufacturer’s instructions. Complementary mutagenic primers, including the mutated regions 5'-CCTGAGTGAGGGGGGTATTAAGCCCAGCCAGTGTTTTTTGTTGTCTCCAA-3' (forward) and 5'-TTGGAGACAACAAAAAACACTGGCTGGGCTTAATACCCCCCTCACTCAGG-3' (Reverse) were designed by the web-based QuikChange Primer Design Program ([www.agilent.com/genomics/qcpd](http://www.agilent.com/genomics/qcpd)). The fidelity of the cloning and mutagenic procedure was checked by sequencing the entire constructs at the Genomic Facilities of Institute of Biomedical Research (IIBm; Madrid, Spain).

# Luciferase assay. To measure the luciferase activity upstream of DICER1 3'UTR, the pIS1 DICER1 long UTR vector (WT) or the same vector containing a mutation in the miR-146b binding site (MUT) was transfected together with pCMV-*Renilla* and the corresponding miRNA expression vector. At 48 h after transfection, cells were harvested and assayed with the Dual-Luciferase Reporter Assay Kit (Promega Corporation). Luciferase activity was normalized to *Renilla* activity.

**Immunoblotting and immunohistochemistry.** Protein concentration was measured by the Bradford method using an assay from Bio-Rad Laboratories. Samples were separated by SDS-PAGE and transferred to nitrocellulose membranes (Bio-Rad). Immunohistochemistry was performed as described (Riesco-Eizaguirre *et al*. Endocr. Relat. Cancer; 13: 257–69, 2006) in tissues previously fixed in 10% buffered formaldehyde for 24 h, embedded in paraffin, and cut into 6-μm serial sections. The following antibodies were purchased from Santa Cruz Biotechnology Inc. (Santa Cruz): β-actin (sc-1616R), TWIST (sc-15393), ZEB1 (H-102), fibronectin (sc-71113), N-cadherin (sc-393933) and tubulin (sc-5286). DICER 13D6 (ab14601) and PCNA (ab92552) antibodies were purchased from Abcam.

**Cell proliferation assays.** To determine cell proliferation by crystal violet staining, 2 × 104 cells were seeded in each well of a 12-well plate 24 h after transfection. Individual wells were fixed in 4% formaldehyde after 48 and 72 h, stained with crystal violet, and cells were counted using ImageJ (NIH). DNA synthesis was determined using a chemiluminescence-based cell proliferation ELISA BrdU assay from Sigma-Aldrich (#11669915001). Cells were seeded in 96-well plates (1 × 103 or 2 × 103 cells/well), pulse-labeled for 4 h with 10 μM BrdU, and measurements were carried out in a luminometer (Promega).

**Migration assay.** Wound healing assays were performed on 90% confluent cell monolayers. Twenty-four hours after transfection, cells were treated for 2 h with 10

µg/mL mitomycin C in 10% fetal bovine serum (FBS) in medium to inhibit proliferation. After treatment, monolayers were scratched using a 10 µL pipette tip and the width of the wound was measured at the described times.

**Invasion assay.** Cells at 3 × 104 (TPC1 and Cal62) or 5 ×104 (SW1736) were suspended in culture medium with 0.2% FBS and placed in the upper chamber 24 h after transfection. The top filter was soaked with 5% FBS in medium 2 h before adding the cells and the lower chamber contained 0.75 mL of medium with 20% FBS as a chemoattractant. Cells were allowed to invade for 24 h at 37ºC and 5% CO2. Non- invading cells in the upper chamber were removed with a cotton swab and membranes were fixed in 4% formaldehyde in PBS and stained with crystal violet. The total number of cells invading the lower surface was counted using ImageJ. Ten fields for each condition were quantified.

***In vivo* studies.** Orthotopic implantation was established in 7-week-old female BALB/c nu/nu mice by thyroid injection in the right lobule with 500,000 or 100,000 Cal62-Luc cells suspended in 5 μL PBS for enoxacin or the anti-146b treatments, respectively. Tumors were generated 2–3 weeks after cell injection, and the bioluminescent signal was detectable. Mice were randomly divided in two groups with similar tumor size and treated by intraperitoneal administration of 15 mg/kg enoxacin (diluted in PBS with 5% DMSO) (n=8) or an equivalent control solution (n=7), every day for 30 days. For anti-146b treatment, mice were treated systemically by administration through the retro-orbital vein with 7 nmol of has-miR-146b-5p mirVana® miRNA inhibitor (n=8) or negative control #1 (n=5) in the transfection reagent Invivofectamine 3.0 (all from Thermo Fisher), 3 times/week for 2 weeks.

Tumor bioluminescent signals were determined *in vivo* twice weekly to calculate tumor growth. To do this, 50 μL of a 40 mg/mL solution of XenoLight D-Luciferin- K+ Salt Bioluminescent Substrate (Perkin Elmer) was injected subcutaneously into each mouse at each time point. At 8 minutes post-injection, mice were anesthetized and imaged using the IVIS-Lumina II Imaging System (Caliper Life Sciences). After 30 days of treatment, tumors were sectioned and fixed for immunohistochemistry. Liver was taken and stained with hematoxylin and eosin and analyzed by a pathologist from the Anatomy Department of Mostoles Hospital (Madrid, Spain).

**Glucose assay.** Serum was collected from mice at the end of the different treatments. Glucose levels in the serum were analyzed using the Glucose Oxidase/Peroxidase Kit (Biosystems) according to the manufacturer’s instructions. Briefly, 10 µL of each sample were added to 250 µL of the reactive solution in a 96-well plate. After 30 min incubation at 37ºC the absorbance at 505 nm was measured.
