# Supplementary figures and images for: Impaired microRNA processing by DICER1 downregulation endows thyroid cancer with increased aggressiveness
Source: Oncogene. 2019 Apr 9;38(27):5486–99. doi: 10.1038/s41388-019-0804-8 (PMC6755984; doi:10.1038/s41388-019-0804-8)

a

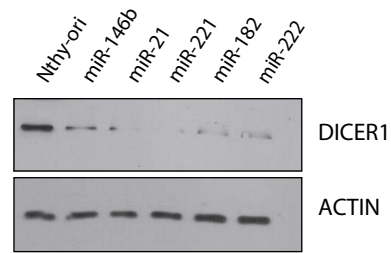

b

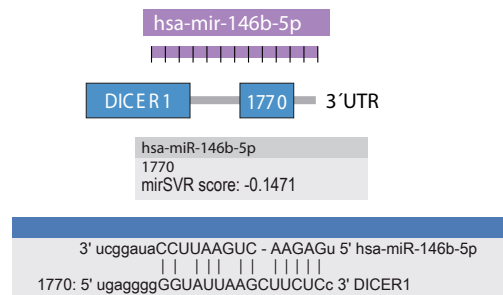

c

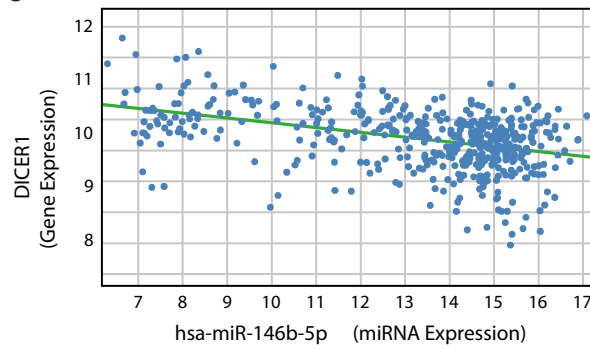

d

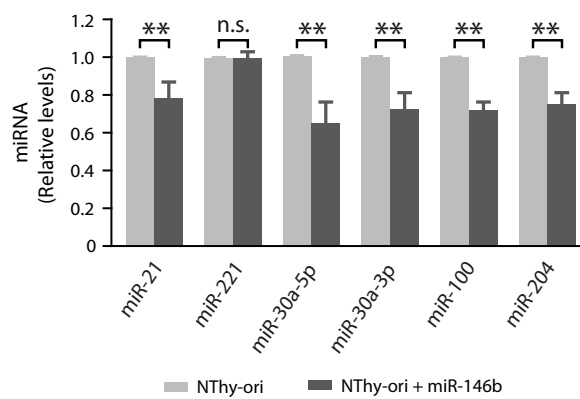

Supplement: Supplementary file 2 — Figure S1 [file 41388_2019_804_MOESM2_ESM.pdf]

**a**

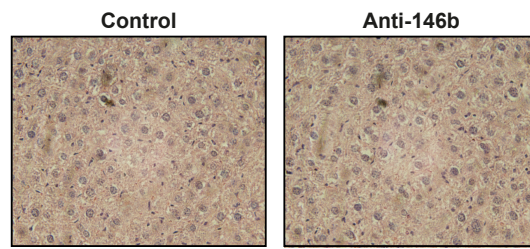

**b**

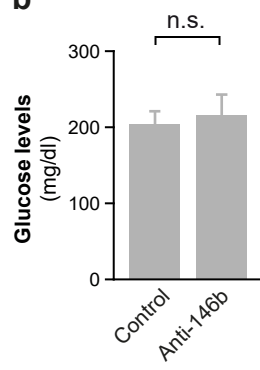

**c**

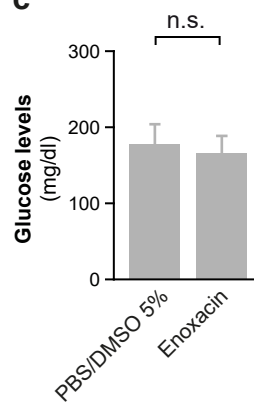

Supplement: Supplementary file 3 — Figure S2 [file 41388_2019_804_MOESM3_ESM.pdf]

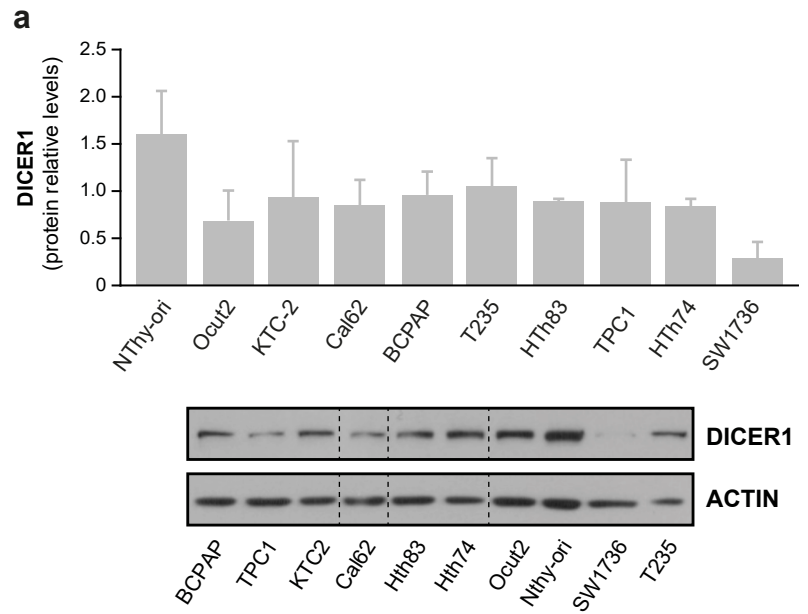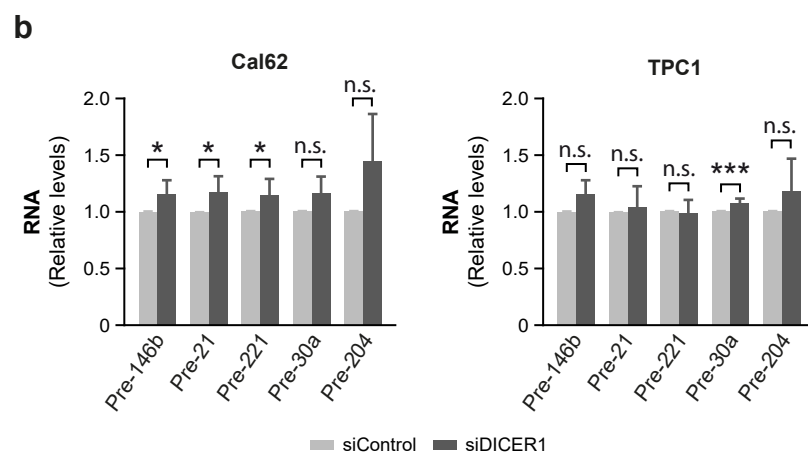

Supplement: Supplementary file 4 — Figure S3 [file 41388_2019_804_MOESM4_ESM.pdf]

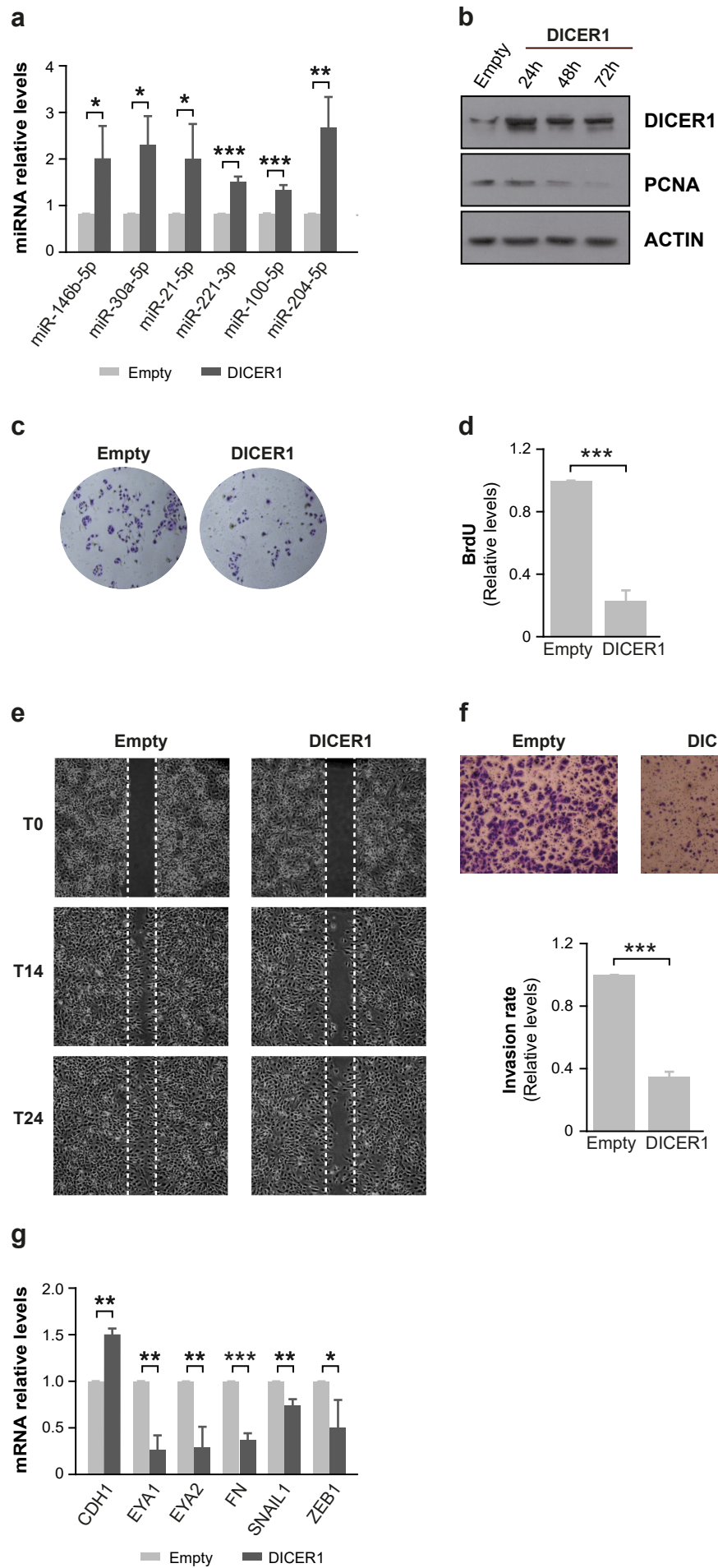

Supplement: Supplementary file 5 — Figure S4 [file 41388_2019_804_MOESM5_ESM.pdf]

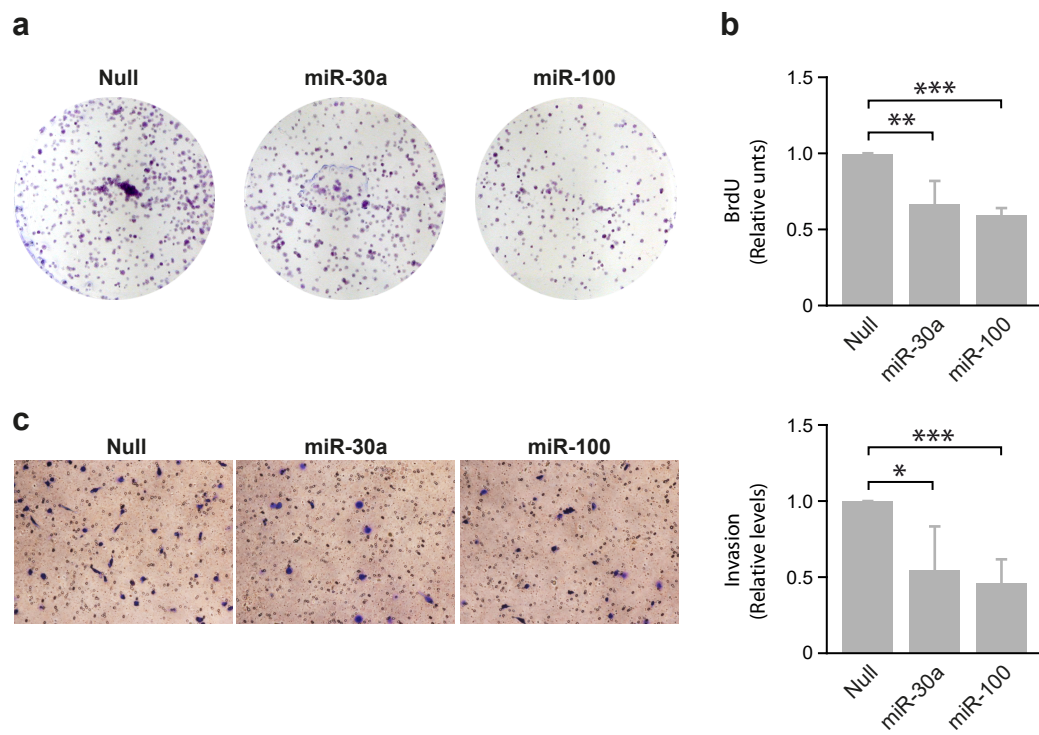

Supplement: Supplementary file 7 — Figure S6 [file 41388_2019_804_MOESM7_ESM.pdf]

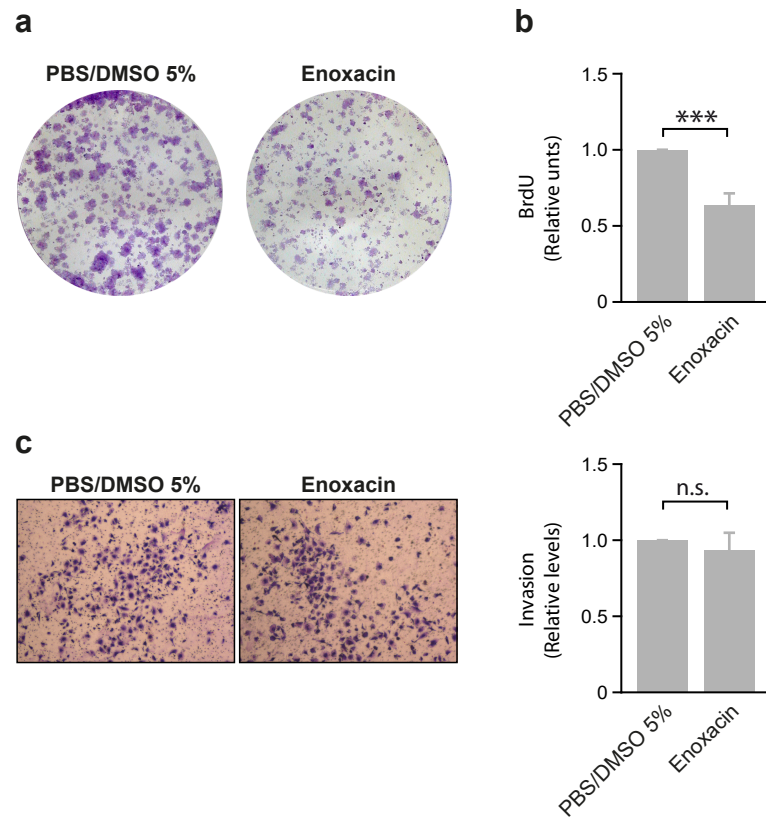

Supplement: Supplementary file 8 — Figure S7 [file 41388_2019_804_MOESM8_ESM.pdf]

**a**

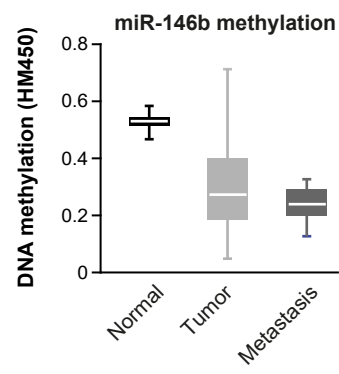

Supplement: Supplementary file 9 — Figure S8 [file 41388_2019_804_MOESM9_ESM.pdf]
