# Supplementary material for: Impaired microRNA processing by DICER1 downregulation endows thyroid cancer with increased aggressiveness
Source: Oncogene. 2019 Apr 9;38(27):5486–99. doi: 10.1038/s41388-019-0804-8 (PMC6755984; doi:10.1038/s41388-019-0804-8)

a

Cluster 1	miR-182, miR-183, miR-204 (high)	Low risk
Cluster 2	miR-142, miR-143	Low risk
Cluster 3	miR-148a, miR-142	Low risk
Cluster 4	Lel7a, Let7f, Let7e, Let7b.	Low risk
Cluster 5	miR-146b-5p, miR-146b-3p, miR-375, miR-221, miR-222, miR-204 (low)	High risk
Cluster 6	miR-21, miR-221, miR-222, miR-204 (low)	High risk

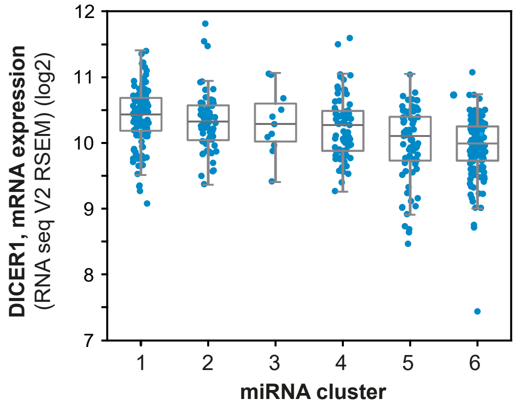

b

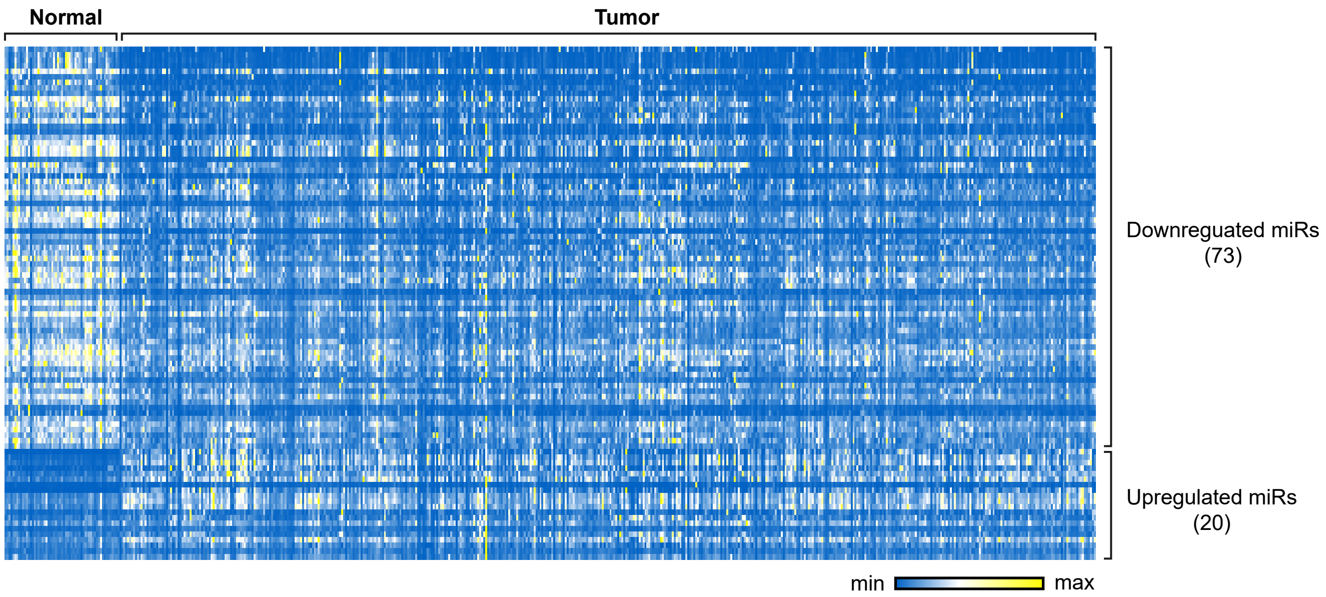

Supplement: Supplementary file 6 — Figure S5 [file 41388_2019_804_MOESM6_ESM.pdf]
