# Supplementary material for: Impaired microRNA processing by DICER1 downregulation endows thyroid cancer with increased aggressiveness
Source: Oncogene. 2019 Apr 9;38(27):5486–99. doi: 10.1038/s41388-019-0804-8 (PMC6755984; doi:10.1038/s41388-019-0804-8)
Supplement: Supplementary file 10 — Suppl. tables [file 41388_2019_804_MOESM10_ESM.docx]

**Supplementary Tables ONC-2018-01823-RR**

**TABLE SI**. Patients clinic-pathological characteristics.

| **Patient ID** | **Gender** | **Age at diagnosis** | **Diagnosis** | **TNM** | **Extrathyroidal extension** | **Vascular invasion** | **Total thyroidectomy** |
| --- | --- | --- | --- | --- | --- | --- | --- |
| 1 | M | 67 | PTC Classic | T2N1aM0 | NO | NO | YES |
| 2 | M | 40 | PTC Classic | T1bN1aM0 | NO | NO | YES |
| 3 | M | 50 | PTC Classic | T1bNxM0 | NO | NO | YES |
| 4 | M | 59 | FTC Oncocytic Variant | T4bNxM0 | YES | YES | YES |
| 5 | F | 33 | PTC Follicular Variant | T1bNxM0 | NO | NO | YES |
| 6 | F | 44 | PTC Classic | T1aNxM0 | NO | NO | YES |
| 7 | M | 28 | PTC Classic | T3N1bM0 | YES | YES | YES |

**TABLE SII**. Primers

PRIMERS I: Coding genes

| **GENE** | **FORWARD PRIMER** | **REVERSE PRIMER** |
| --- | --- | --- |
| DICER1 | GGTGGTCCACGAGTCACAAT | TAGCACTGCCTTCGTTTCGT |
| ACTIN | CACTCTTCCAGCCTTCCTT | CTCGTCATACTCCTGCTTGCT |
| U6 | CTCGCTTCGGCAGCACA | AACGCTTCACGAATTTGCGT |
| NKX2.1 | GCAACGGCAACCTGGGCAAC | ATGAAGCGGGAGATGGCGGG |
| PAX8 | CAAGGTGGTGGAGAAGATTG | GAGGTTGAATGGTTGCTG |
| FOXE1 | TGAGCCAGCGTAGGGACGAAAA | CCACCTCCTCCCGTTTACAGAGTA |
| NIS | GTGGTCTGGACTGATGTGTTC | TGCTGAGGGTGCCACTGTA |
| CDH1 | CAGCACGTACACAGCCCTAA | ACCTGAGGCTTTGGATTCCT |
| EYA1 | AGCCTGGCTGCATTGAGGGCC | AGTCGGTCAGGGCTTCAATTT |
| EYA2 | TAAACCTCATCAACTCCCGGCCCAA | GGTGACCAGCACATTGACACA |
| FIBRONECTIN | CCCTTACAGTTCAGGGTTCC | TTCAAGCCTTCGTTGACAGA |
| PAI1 | TCTCTGCCCTCACCAACATTC | ACATGTCGGTCATTCCCAGGTTCT |
| SNAIL1 | CTCTTTCCTCGTCAGGAAGC | GGCTGCTGGAAGGTAAACTC |
| TWIST1 | GGCCGGAGACCTAGATGTCATTGTT | GCCCCACGCCCTGTTTCTTTGAAT |
| ZEB1 | GCCAATAAGCAAACGATTCTG | TTTGGCTGGATCACTTTCAAG |

PRIMERS II: miRNAs

| **miRNA** | **PRIMER** |
| --- | --- |
| miR-221-3p | AGCTACATTGTCTGCTGGGTTTC |
| miR-30a-5p | TGTAAACATCCTCGACTGGAAG |
| miR-21-5p | TAGCTTATCAGACTGATGTTGA |
| miR-146b-5p | TGAGAACTGAATTCCATAGGCT |
| miR-100-5p | AACCCGTAGATCCGAACTTGTG |
| miR-204-5p | TTCCCTTTGTCATCCTATGCCT |
| miR-30-3p | CTTTCAGTCGGATGTTTGCAGC |

PRIMERS III: pre-miRNAs

| **Pre-miRNA** | **FORWARD PRIMER** | **REVERSE PRIMER** |
| --- | --- | --- |
| Pre-miR-146b | TGGCACTGAGAACTGAATTCCATG | CCGGGCACCAGAACTGAGTC |
| Pre-miR-21 | TGTCGGGTAGCTTATCAGACTGA | TGTCAGACAGCCCATCGACTGGT |
| Pre-miR-221 | TGAACATCCAGGTCTGGGGCAT | GAGAACATGTTTCCAGGTAGCCT |
| Pre-miR-30a | GCGACTGTAAACATCCTCGACT | GCAGCTGCAAACATCCGACTGA |
| Pre-miR-204 | GGCTACAGTCTTTCTTCATGTG | GCCAGTGATGACAATTGAACGTC |
