# Supplementary material for: Impaired microRNA processing by DICER1 downregulation endows thyroid cancer with increased aggressiveness
Source: Oncogene. 2019 Apr 9;38(27):5486–99. doi: 10.1038/s41388-019-0804-8 (PMC6755984; doi:10.1038/s41388-019-0804-8)
Supplement: Supplementary file 11 — Suppl. Figure Legends [file 41388_2019_804_MOESM11_ESM.docx]

**Supplementary (S) Legends of Figures and Tables.**

**Figure S1.** **The major upregulated miRNAs including miR-146-5p for the thyroid cancer target DICER1.** **(A)** Immunoblot of DICER1 in Nthy-ori cell lines 48 h after transient transfection with miR-146b, miR-21, miR-221, miR-182, miR-222, or the empty vector. Actin was used as a loading control. **(B)** Top: schematic representation of miR-146b-5p and the predicted binding site in the DICER1 3’UTR, represented as a blue box. Bottom: mirSVR score and duplex formation between human DICER1 3′UTR and miR-146b-5p predicted by miRanda. **(C)** Correlation between DICER1 and miR-146b-5p using Cancer Regulome analysis in the TCGA database (Correlation = -0.39, adj. p-value>0.001). **(D)** Relative expression levels of miR-21, miR-221, miR-30a, miR-30a-3p, miR-100 and miR-204 in the Nthy-ori cell line transfected with miR-146b or the empty expression vector. Values represent mean ± SD (*n* = 3). **p< 0.01; n.s non-significant.

**Figure S2. The systemic administration of anti-miR146b or enoxacin has no severe toxic effects. (A)** Hematoxylin-eosin staining of liver slices from mice treated with the miR-146b inhibitor (Anti-146b) or the control. **(B, C)** Glucose levels (mg/dL) in control mice or in mice treated with (B) the miR-146 inhibitor or (C) with enoxacin. n.s non-significant.

**Figure S3. Analysis of DICER1 and pre-miRNAs**. **(A)** Quantification of DICER1 protein in the indicated thyroid cell lines (top) and a representative immunoblot (bottom). Actin was used as a loading control. **(B)** Relative levels of pre-146b, pre-21, pre-221, pre-30a and pre-204 in Cal62 and TPC1 cell lines silenced for DICER1, analyzed by RT-qPCR. Values represent mean ± SD (*n* = 3). *p< 0.05; ***p< 0.001; n.s non-significant.

**Figure S4**. **DICER1 overexpression decreases proliferation, migration and invasion, and EMT markers.** Gain-of-function assays were performed in SW1736 cells. **(A)** Expression levels of miR-221-3p, miR-30a-5p, miR-21-5p, miR146b-5p, miR-100-5p, miR-204-5p and miR-451a 48 h after DICER1 (or empty vector) transfection. **(B)** Immunoblot of DICER1, PCNA and actin, used as a loading control. **(C)** Representative crystal violet-stained images of SW1736 cells 48 h after DICER1 transfection. **(D)** BrdU incorporation relative to cells transfected with the empty vector. **(E)** Representative images from a wound healing assay 0, 14 and 24 h after scratching. **(F)** Quantification of invasion rates. Top: representative images of the lower chamber (invading cells). Bottom: cell invasion rates relative to the empty vector-transfected cells. **(G)** mRNA relative levels of EMT genes *CDH1*, *EYA1*, *EYA2*, *FN*, *SNAIL1* and *ZEB1* 48 h after DICER1 transfection. Values represent mean + SD (n = 3). *p<0.05; **p<0.01; ***p<0.001.

**Figure S5. TGCA data analysis of DICER1 levels in miRNA clusters and differential expression of miRNAs**. (A) Left: table adapted from Riesco-Eizaguirre and Santisteban Eur. J Endocrinol 175: R203-217, 2016) of miRNA clusters. Right: correlation between DICER1 mRNA and miRNas analyzed using the cBioPortal of TCGA (adjusted p-value<0.001). (B) The heatmap represents the differentially expressed miRNAs analyzed by TCGA. False Discovery Rate (FDR) <0.01 and fold change (FC) >2 for the upregulated miRs and FC <0.75 for those downregulated.

**Figure S6. miR-30a and miR-100 decrease proliferation and invasion in Cal62 thyroid cells. (A)** Representative crystal violet-stained images of Cal62 cells 96 h after miR-30a or miR-100 transfection. **(B)** BrdU incorporation relative to cells transfected with the empty vector (Null). **(C)** Quantification of invasion rates. Left: representative images of the lower chamber (invading cells). Right: cell invasion rates relative to the empty vector-transfected cells (Null). Values represent mean + SD (n = 3). *p<0.05; **p<0.01; ***p<0.001.

**Figure S7. Enoxacin treatment in the Nthy-ori thyroid cell line. (A)** Representative images of crystal violet-stained Nthy-ori cells 5 days after 5% PBS/DMSO or enoxacin treatment. **(B)** BrdU incorporation relative to cells treated with 5% PBS/DMSO. **(C)** Quantification of invasion rate. Left: representative images of the lower chamber (invading cells). Right: cell invasion rates relative to the 5% PBS/DMSO treatment. Values represent mean ± SD (*n* = 3). ***p< 0.001; n.s non-significant.

**Figure S8. miR-146b methylation levels in normal tissue, PTC patients and metastasis.** (A) Box plot of miR-146b methylation in normal thyroid tissue, PTC, and metastases: data was obtained from the TCGA database.

**Supplementary Table I. Patient clinical details.**

**Supplementary Table II.** Primer list for coding genes and miRNAs and pre-miRNAs
